# Research on the effect of different aerobic activity on physical fitness and executive function in primary school students

**DOI:** 10.1038/s41598-024-58009-7

**Published:** 2024-04-04

**Authors:** Yue Ren, Jun Chu, Zhongyuan Zhang, Bingquan Luo

**Affiliations:** 1 Capital University of Physical Education and Sports, Beijing, 100191 China; 2 College of Art and Design, Zhengzhou University of Economics and Business, Zhengzhou, 451100 China

**Keywords:** Different aerobic activity, Physical fitness, Cognition, Students, Physiology, Psychology, Environmental sciences, Environmental social sciences

## Abstract

To evaluate the effect of 16 weeks of different aerobic activity on physical fitness and executive functions in primary school students. 90 right-handed students from China (Boys = 46; Girls = 44) participated in our study and were randomly separated into four groups: 20 in the control group (Con), 23 in the physical activity group (PA), 25 in the intellectual activity group (IA), 22 in the physical activity and intellectual activity group (PA + IA). The students in PA, IA and PA + IA group received aerobic exercise program lasted 40 min daily, 4 days a week for 16 weeks, regular physical activity in the PA group, intellectual activity in the IA group, physical activity couple with intellectual activity in the PA + IA group, respectively. All the students participate the experiment for body composition, physical fitness (cardiopulmonary fitness, muscle strength, speed sensitivity, flexibility quality), executive functions and saliva analysis test before and after 16 weeks. There was no significant effect of 16 weeks different aerobic exercise interventions on body composition before and after exercise interventions among four groups in children (p > 0.05). The results were obtained by inter-group and intra-group comparisons that different exercise interventions (physical activity, intellectual activity, physical combine with intellectual activity), all can significantly improve physical fitness parameters (cardiopulmonary fitness, muscle strength, speed sensitivity and flexibility quality), and executive functions parameters (inhibitory control, working memory, reaction time cognitive flexibility), as well as the concentration of saliva GH and IGF-I (p < 0.05) in children. Our experiment further demonstrated that the improvement effect of the two exercises together is more significant than that of the single exercise ways. Both physical and intellectual activity can effectively improve physical fitness and executive function in children, and the improvement effect of the two exercises together is more significant than that of the single exercise ways.

## Introduction

With the continuous reform of school education in recently years, society pays more attention to the all-around development of students, especially in primary school stage^1^. As a critical period of growth and development, student’s physical and mental health status in primary school directly affects the development of adolescents and even adults. On the one hand, physical fitness (PF) can be considered as the comprehensive performance of physical functions related with lifestyle, which consists of multiple components such as cardiorespiratory fitness, musculoskeletal fitness (i.e., muscular power, endurance, and flexibility), agility, speed^2^. There are a number of cross-sectional studies and systematic reviews showing that the trend of continuous decline in children’s PF has increased alarmingly, which may affect their health, educational level, and quality of life. On the other hand, executive function (EF) is defined as a set of higher-order neurocognitive domain component processes, such as inhibitory control, working memory, reaction ability, shifting^3^. Increasingly empirical evidence focus on the crucial role of executive function on children’s academic and behavioral beginning as early as young childhood^4^. However, deficits of executive functions in childhood deeply influence academic achievement and daily life, even producing of great etiologically significance for a broad range of brain diseases, eg. depression^5^, attention deficit hyperactivity disorder^6^, bipolar disorders^7^. Therefore, children are at a stage of great plasticity, it is necessary to find an economical and effective way for developing PF and EF at child stage.

Research on positive effect of exercise in early childhood has flourished in recent years. Exercise has been proposed as a low-cost and safe intervention method for the general child population, with a variety of favorable physiological, psychological, and neurocognitive impacts^8^. One is, exercise in the anthropometrical and physiological aspects of positive effect. Various report have shown that long term exercise can control weight gain^9^, promote the blood circulation^10^, improve physical fitness (e.g., cardiopulmonary fitness, muscle strength, speed sensitivity and flexibility quality)^11^. Two is, exercise in the psychological aspects of positive effect. Exercise might act as an endogenous stimulus to release mental stress eventually leading to reduce mental disorder in childhood, even in the whole lifespan. These include reductions in stress^12^, anxiety^13^, depression^14^ and negative affect^15^. Three is, exercise in the neurocognitive and biomechanical aspects of positive effect. Exercise may operate as a neuro-enhancer and increase cognitive performance by influencing neurotransmitter transmission, neurotrophic signaling, and neuroplasticity pathways in the short and long term, such as working memory, inhibitory control, shifting^16,17^. As discussed below, exercise that is both coordinative and challenging may be more engaging and efficient. As an example, Diverse activities, such as computerized training, non-computerized games, aerobics, yoga, mindfulness, and school exercise curricula, have all been found to improve children's executive function^18^. It is, however, noteworthy there are only a few literatures to reveal the effect of different exercise on children. More studies are thus needed in particular in children detailing the influence of different exercise. Based on the above, this study aimed to explore the effect of simultaneous intervention of physical activity and intellectual activity on children.

## Methods

### Study population

**Figure 1:**
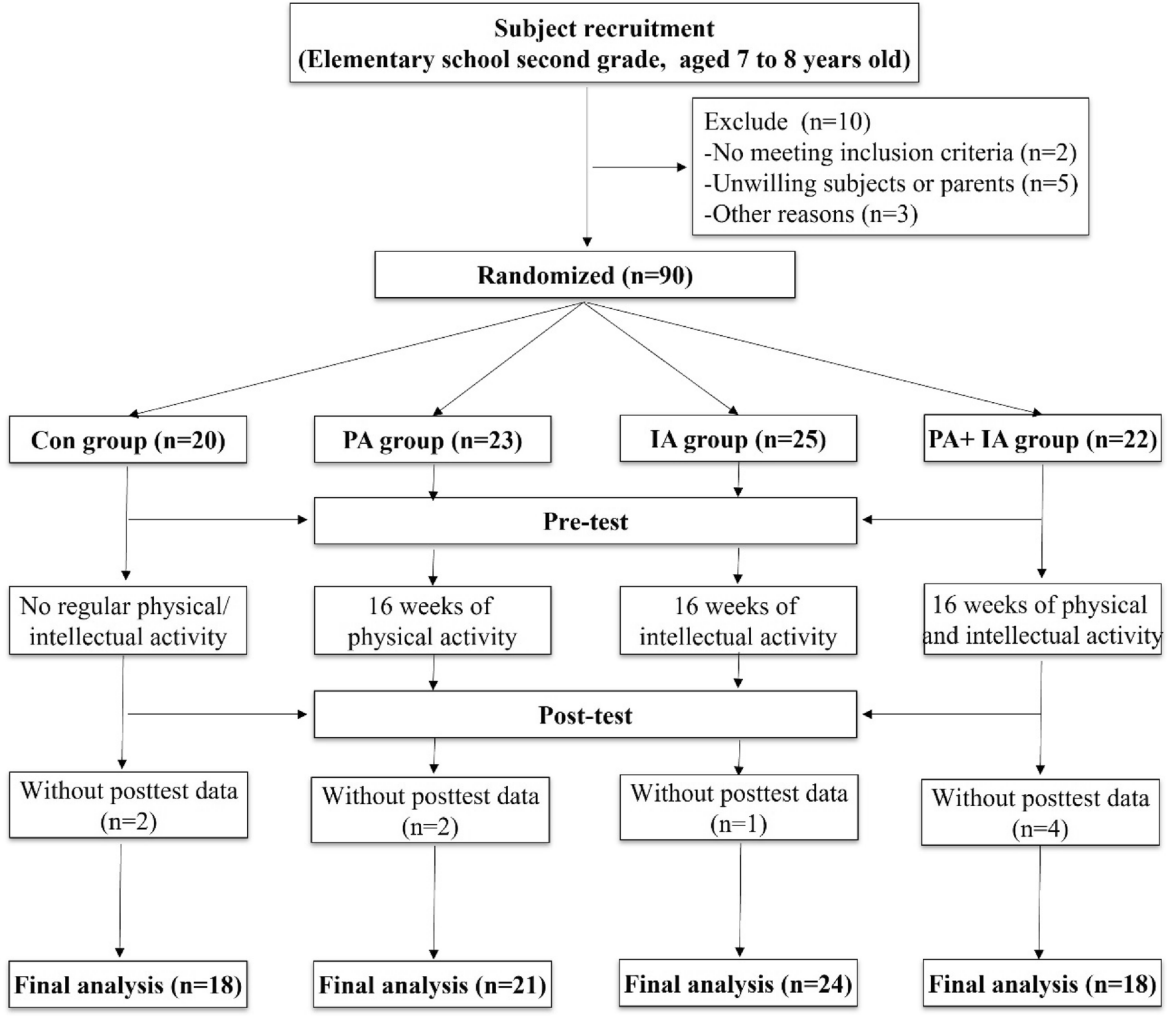
Participant flowchart across the study.

Sixty-five healthy students (Boys = 32; Girls = 33; 7-year-olds) in primary school-Beijing were recruited for this single-blind randomized trial (Fig. 1). The students all went to the same school and had normal neurological function. Inclusion criteria: (1) who were 7 to 8 years old, (2) had no surgery or drug histories in the previous 1 year, (3) did not participate in other organized physical exercise in their spare time, and (5) whose parents/guardians agreed to participate in the study. The study design and protocol were in line with the Declaration of Helsinki and was approved by Capital University of Physical Education and Sports (2020A97).

Finally, 90 right-handed youngsters from China (Boys = 46; Girls = 44) participated in our study and were randomly separated into four groups: Control group (Con group, n = 20), physical activity group (PA group, n = 23), intellectual activity group (IA group, n = 25), physical activity group and intellectual activity group (PA + IA group, n = 22). They all followed a systematic physical activity intervention excepted con group for 11 weeks. Before and after the 11-week exercise intervention, they were evaluated on participant characteristics, physical fitness, and executive functioning. At the end of the trial, 81 children finished it, and their results were considered effective samples (Con group, n = 18; PA group, n = 21; IA group, n = 24; PA + IA group, n = 18; Fig. 1).

### Aerobic exercise interventions

In this study, the students in the PA, IA and PA + IA group received aerobic exercise program lasted 40 min daily, 4 days a week for 16 weeks, regular physical activity in the PA group, intellectual activity in the IA group, physical activity couple with intellectual activity in the PA + IA group, respectively.

**Figure 2:**
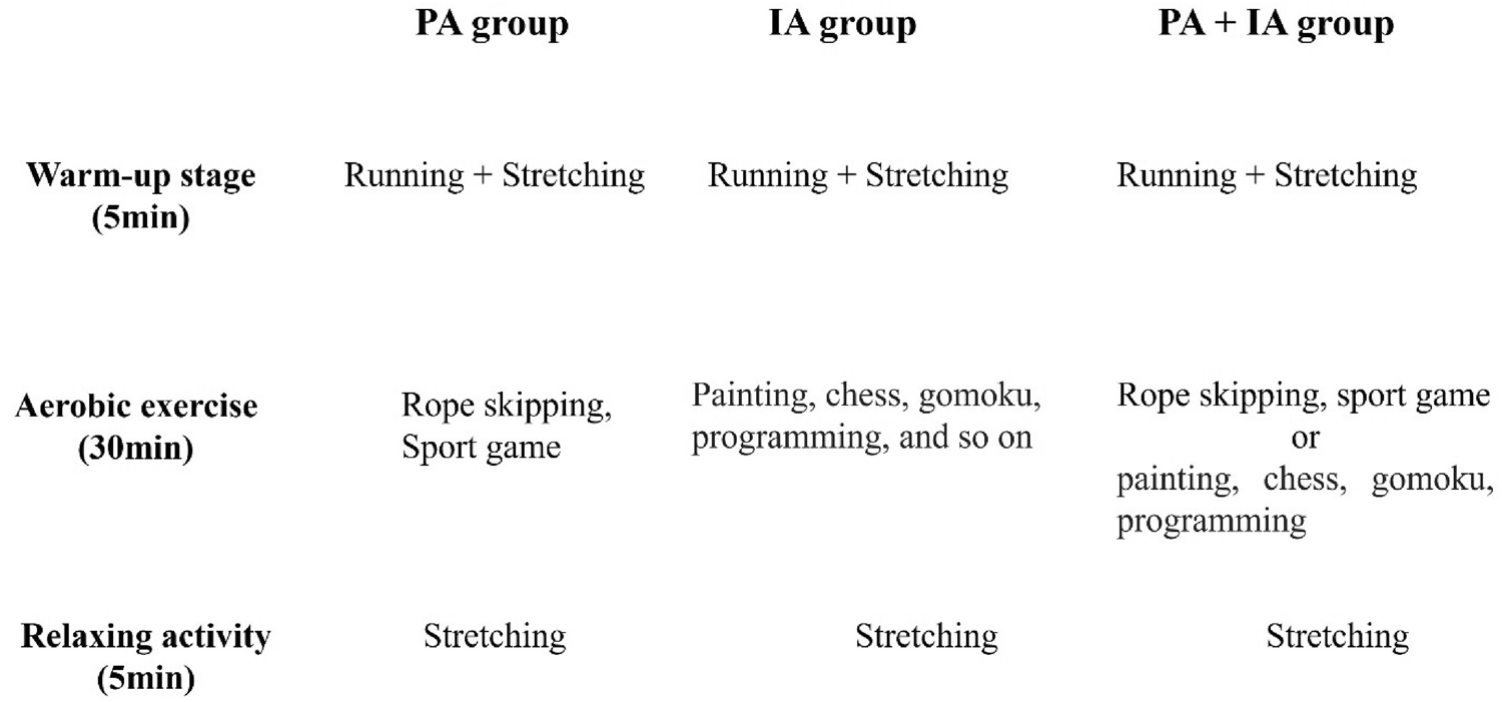
Exercise training protocol.

The 16-week physical exercise comprised 3 sessions for 40 min each. The experimental intervention took place every week 1 and 3 from 15:50 to 17:50 and was performed by trained physical education teacher. An example of the physical exercise lesson is shown in Fig. 2. And physical exercise is different, intellectual activity mainly including painting, chess, gomoku, programming, and so on.

### Anthropometric

The participant’s standing height and body weight were measured barefoot using a Holtain stadiometer and Inbody J10 (Biospace Corp., Seoul, Korea) and portable Tanita scale (Model BF-522; Tanita Corporation, Tokyo, Japan). Weight and height were calculated to the closest 0.1 kg and 0.1 cm. BMI values were translated into percentiles (weight in kilos divided by height in meters squared)^19^.

### Physical fitness

The participant’s physical fitness was evaluated through a series of experiments^20^:

#### Cardiopulmonary fitness: 50-m run

In this test, the students stand in front of the white starting line, ready to prepare running posture. When all was ready a signal of “Get ready! Set! Go!” was given, the students were encouraged to run as far until they finished 50 m. the time spend in this task were recorded.

#### Muscle strength: handgrip strength and standing long jump

In handgrip strength test, the test will be performed while standing, with the wrist in a neutral posture and the elbow extended. Children will be encouraged exert maximum effort for at least 3 s. The best of the two values (kg) will be picked, and the average of both hands will be recorded. In standing long jump test, the child stood with feet shoulder width apart and jumped as far as they could. The distance is calculated, and the best of these attempts being recorded.

#### *Speed sensitivity: 4* × *10 m shuttle run*

On the floor, two parallel lines 10 m apart will be drawn using marking tape. Children were encouraged to sprint and turn between the two lines as quickly as possible until they could no longer run the 20-m distance or stopped due to exhaustion. The distance covered by the test is 40 m. The number of laps completed will be used to indicate test results, and the best of two attempts will be recorded.

#### Flexibility quality: sit and reach

The participants were told to sit on the floor with their legs outstretched in front of them. Both of knees were parallel to the ground. Participants extended their upper limbs as far as they could, with elbows extended, hands on top of each other, and palms facing down along the measurement line. The best of three recordings was chosen for examination.

### Executive function test

#### Inhibitory control—animal Go/No-go task

The task comprised eight animals, each of which was randomly displayed above the green button. When the animal was not a pig, the green button was swiftly pressed. When the pig appeared, there was no button-pressing response, and only one animal was shown at a time for a maximum of 3 s. This was done 40 times. The appropriate rate was recorded.

#### Working memory—working memory span task

A house was created on the screen, and children were encouraged to remember details about it, such as the names of animals and the shape and color of the windows. Following that, an empty house was projected onto the screen, and the youngsters were grilled regarding its condition. The total number of houses in the experiment increased from one to three.

#### Reaction time—simple reaction test

When the stimuli were displayed on the computer screen, the youngsters were instructed to press the green key as quickly as feasible. We ran 30 of these trials with a 3-s interval.

#### Cognitive flexibility—flexible item selection task

Sort the cards in the flexible item selection task by color, size, or category. Two photographs from the same category were shown on a screen, and the children were questioned about the picture category. Following that, when a new image appeared on the screen, the children were quizzed on the category of the image in relation to the prior ones. Finally, three photographs were shown together, and the youngsters were instructed to select two similar pictures in a specific dimension.

### Sample collection and analysis

Saliva samples were taken 2 days before and 2 days after the physical exercise intervention. The samples were immediately deposited in a 1.5 ml tube and centrifuged at 4 °C before being kept at − 80 °C until analysis. Human Growth Hormone ELISA Kit (EK110, Multi Science) and Human IGF-I ELISA Kit (PI488, Beyotime) were applied to detected the level of growth hormone and insulin-like growth factor I. The double-antibody sandwich ELISA method is used in this kit to quantify human IGF-I in samples. The plate is precoated with monoclonal antibodies against human IGF-I as capture antibodies, and when a standard or sample is introduced, the human IGF-I binds to the capture antibody. The biotin-conjugated human IGF-I antibody is then applied to the plate, where it binds to human IGF-I to create a sandwiched immunological complex. Following that, HRP-labeled Streptavidin is introduced and binds to the sandwich immune complex via the biotin-streptavidin interaction. Finally, TMB Solution is added to start the chromogenic process. Furthermore, the addition of Stop Solution results in a distinct yellow color that absorbs at 450 nm^21^.

### Statistical analysis

The study sample's characteristics are reported as means standard deviations (SDs). For statistical analysis, SPSS 18.0 and GraphPad Prism 8.0 software were utilized. The paired sample t-test was used for within-group comparisons, while the independent samples t-test was utilized for between-group comparisons. Furthermore, the physical fitness and cognition test results were examined using two (OW/CO group) * two (before and after 16 weeks) repeated measures analysis of variance tests. Aerobic exercise was employed as the inter-subject variable, and time was used as the subjects' internal variable. The statistical significance level was chosen at *p* < 0.05.

### Informed consent

Informed consent was obtained from all participators, and their guardians agreed their children to participate in the study.

## Results

### Body composition before and after exercise

Table 1 shows the anthropometric measurements variables for all of the children in each group in the pre-test and post-test stage. There were no significant differences among each group in the pre-test stage, such as height, weight, BMI (p > 0.05). A similar phenomenon was observed in the post-test stage as no significant changes in anthropometric measurements index were observed over time among four groups (p > 0.05). Table 1 also revealed that no remarkable difference of anthropometric measurements variables in each group with the paired sample t-tests by intragroup comparison (p > 0.05). Thus, there was no significant effect of 16 weeks aerobic exercise interventions on body composition in children.

**Table 1:** Demographic characteristic among four group (Mean ± SD).

	Pre-test				Post-test			
	Con	PA	IA	PA + IA	Con	PA	IA	PA + IA
Height [cm]	122.54 ± 3.23	122.87 ± 2.76	122.24 ± 3.42	122.74 ± 2.47	122.765 ± 2.91	122.96 ± 2.20	122.69 ± 3.23	123.04 ± 2.56
Weight [kg]	23.26 ± 1.41	23.10 ± 0.9	23.14 ± 1.06	23.13 ± 1.50	23.5 ± 1.40	23.36 ± 0.77	23.19 ± 0.7	23.25 ± 1.10
BMI [kg/m^2^]	15.53 ± 1.29	15.32 ± 0.81	15.54 ± 1.25	15.37 ± 1.20	15.63 ± 1.28	15.47 ± 0.84	15.44 ± 1.07	15.38 ± 0.98

Con represent control group; PA represent physical activity group; IA represent intellectual activity group; PA + IA represent physical and intellectual activity group.

### Effect of the physical exercise on physical fitness in each group

**Figure 3:**
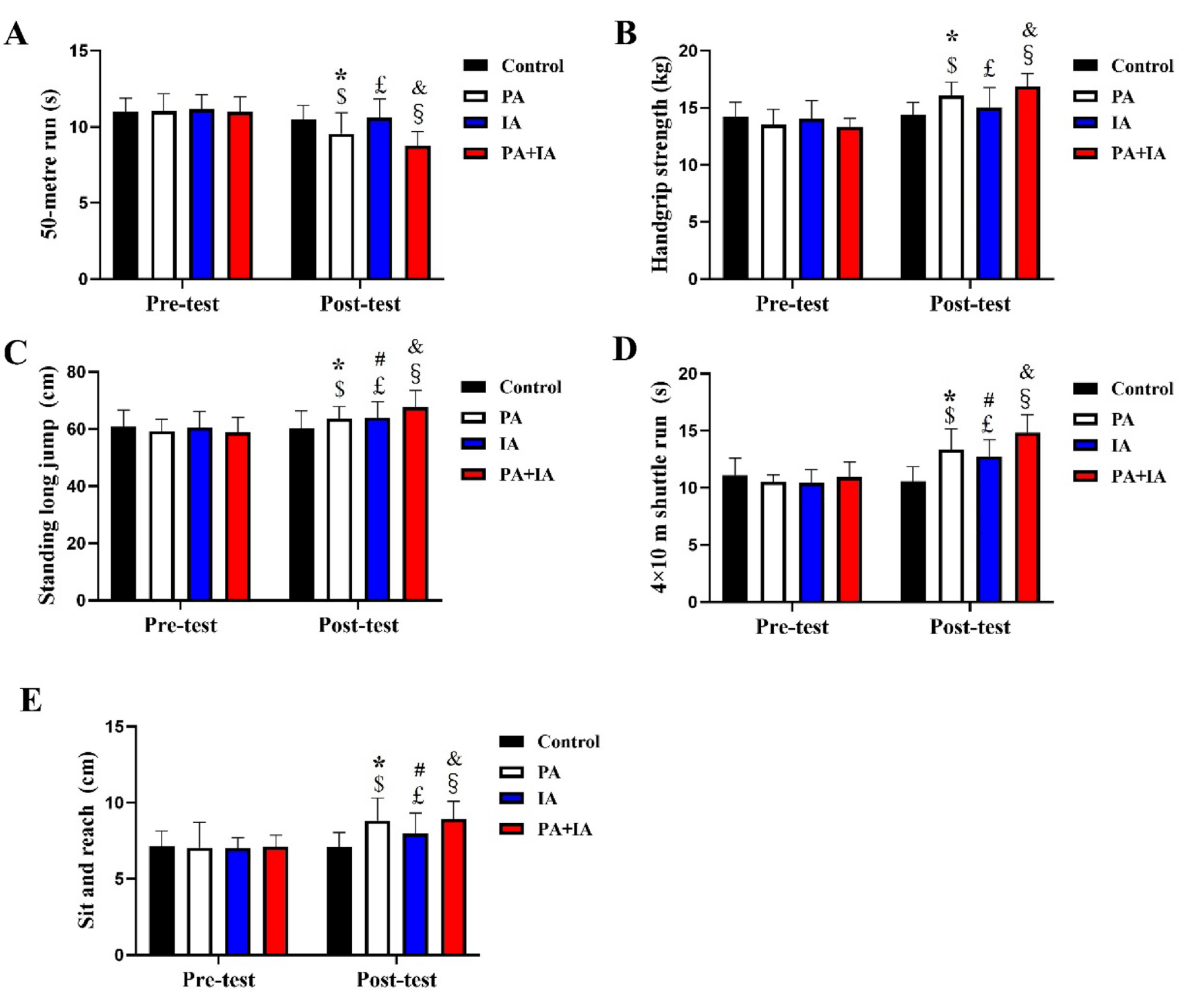
Effect of different physical exercise program on physical fitness in children. The results of upper limb muscle strength in the handgrip strength test (**A**), lower limb muscle strength in the standing long jump test (**B**), cardiopulmonary fitness in the 20-m shuttle run test (**C**), flexibility quality in the sit and reach test (**D**), speed sensitivity in the 4 × 10 m shuttle run test (**E**) in each group. *p < 0.05 represent Con vs. PA; ^#^p < 0.05 represent Con vs. IA; ^&^p < 0.05 represent PA + IA vs. Con. ^$^p < 0.05 represent Pre-test vs. Post-test in the PA group; ^£^p < 0.05 represent Pre-test vs. Post-test in the IA group; ^§^p < 0.05 represent Pre-test vs. Post-test in the PA + IA group; N = 16–24/ per group.

The results of physical fitness tests in each group in the whole experiment can be found in Fig. 3. There were no significant differences among four group in physical fitness parameters (Cardiopulmonary fitness, muscle strength, speed sensitivity, flexibility quality) during the pre-test stage (*p* > 0.05). In the post-test stage, there were significant differences among the groups with in terms of physical fitness parameters (*p* < 0.05). The time spend of the PA + IA and PA groups in the 50 m run test was significantly decreased than that of the IA and Con groups (*p* < 0.05). However, the upper muscle strength of the PA + IA, PA and IA groups in the handgrip strength test significantly higher than in the Con groups (*p* < 0.05). The lower muscle strength of the PA + IA and PA groups in standing long jump test significantly higher than in the other groups (*p* < 0.05). Moreover, speed sensitivity in 4 × 10 m shuttle run and flexibility quality in sit-reach test also showed a significant intergroup difference (*p* < 0.05), and the results showed that the difference was in the order of the PA + IA, PA, IA, Con groups. We can learn that all of the physical fitness variables of the groups were affected by physical activity or/and intellectual activity to some extent, as the effects of the activity intervention were the greatest in the PA + IA group.

Intragroup comparison demonstrated that all the physical fitness parameters represented no significant difference in the Con group between the post- and pre-test (*p* > 0.05). In the PA group, there was significant improvement of physical fitness parameters after 16 weeks physical activity regarding intra-group comparison (*p* < 0.05).

In the IA group, part physical fitness parameters, speed sensitivity and flexibility quality, significantly improved after intellectual activity (*p* < 0.05), but this improvement effect cannot be found in other physical fitness parameters—cardiopulmonary fitness, muscle strength (*p* > 0.05). In the PA + IA group, there was a significant difference of all physical fitness parameters between pre- and post-test (*p* < 0.05).

Based on this, both physical and intellectual activity can effectively improve the physical fitness in children, and the improvement effect of the two exercises together is more significant than that of the single exercise ways.

### Effect of the physical exercise on executive function in each group

**Figure 4:**
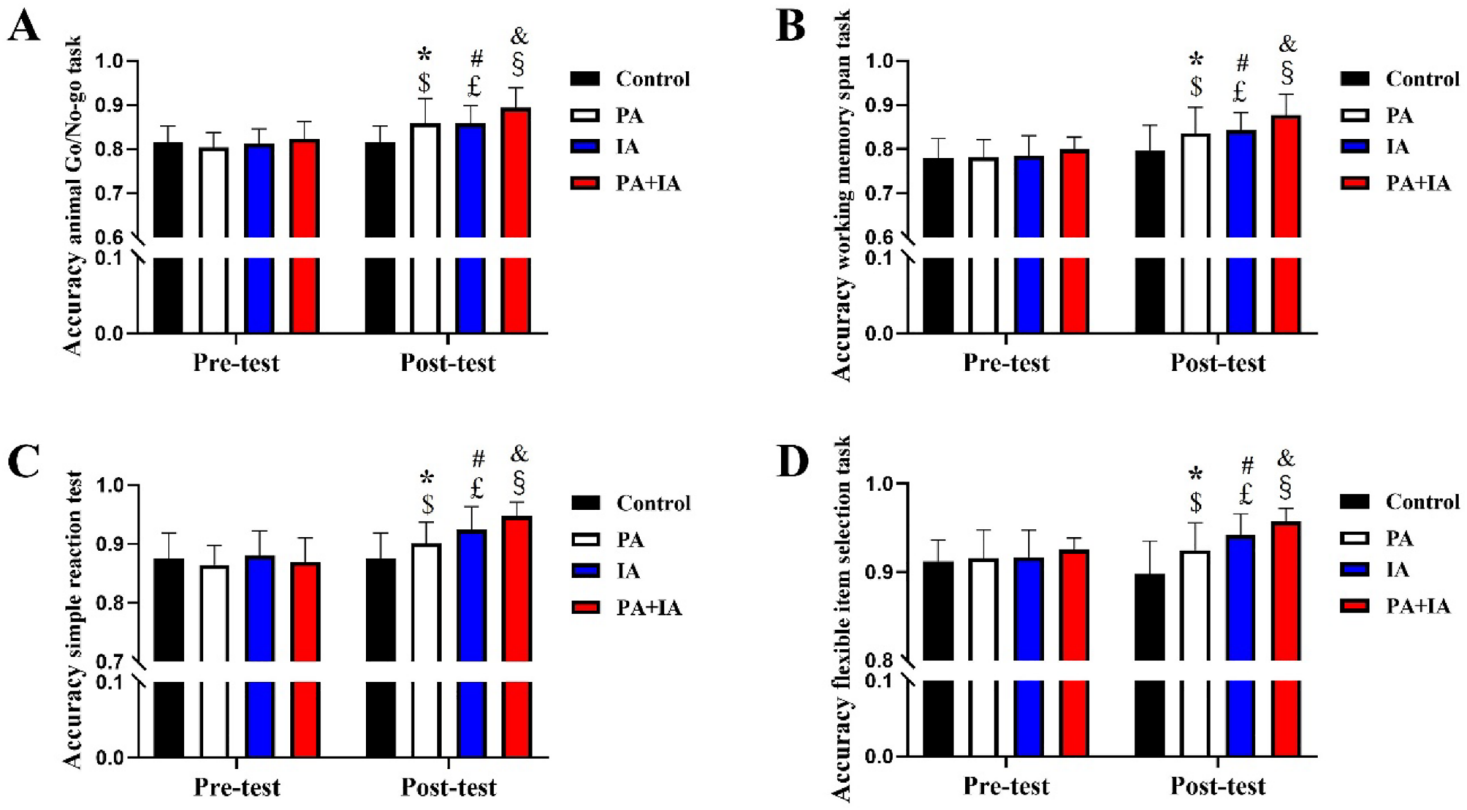
Effect of different physical exercise program on executive function in children. The results of inhibitory control in animal Go/No go task (**A**), working memory in the working memory span task (**B**), reaction time in the simple reaction test (**C**), cognitive flexibility in the flexible item selection task (**D**). *p < 0.05 represent Con vs. PA; ^#^p < 0.05 represent Con vs. IA; ^&^p < 0.05 represent PA + IA vs. Con. ^$^p < 0.05 represent Pre-test vs. Post-test in the PA group; ^£^p < 0.05 represent Pre-test vs. Post-test in the IA group; ^§^p < 0.05 represent Pre-test vs. Post-test in the PA + IA group; N = 16–24/ per group.

In the pre-test stage, there was no significant difference in executive function index among each group in the Fig. 4, including inhibitory control, working memory, reaction time, cognitive flexibility (*p* > 0.05). In order to investigate the effect of different groups aerobic exercise on executive function of children, the mixed variance analysis found that the main effect of exercise intervention of executive function was significant in the post-test stage (*p* < 0.05). A significant difference was observed in inhibitory control, working memory, reaction time among four groups (*p* < 0.05). Post-hoc analysis showed that when compared with Con group, there was a significant difference in the IA group (*p* < 0.01), PA group (*p* < 0.05), PA + IA group (*p* < 0.05), this results also revealed that significantly improvement of inhibitory control, working memory, reaction time in the PA + IA group than that of the PA and IA groups (*p* < 0.05). In addition, we also found that cognitive flexibility presented a significant intergroup difference among each group, and the difference was in the order of the PA + IA, PA, IA, Con groups.

Intra-group comparison: There was no significant difference between the two measurements in the Con group (p > 0.05). In the PA group, the part executive function index of post-test, including reaction time, working memory, was better than pre-test (p < 0.05), but no significantly improvement effect in the remaining executive function index both pre-test and post-test (p < 0.05). The post-test of all the executive function index was better than the pre-test in the IA group (p < 0.05). Similarly, there was a significant improvement of all the executive function index after 16 weeks physical couple with intellectual activity interventions in the PA + IA group (p < 0.05).

It showed that both physical and intellectual activity can effectively improve the executive function in children, and the improvement effect of the two exercises together is more significant than that of the single exercise ways.

### Effect of the physical exercise on GH-IGF-I axis in each group

**Figure 5:**
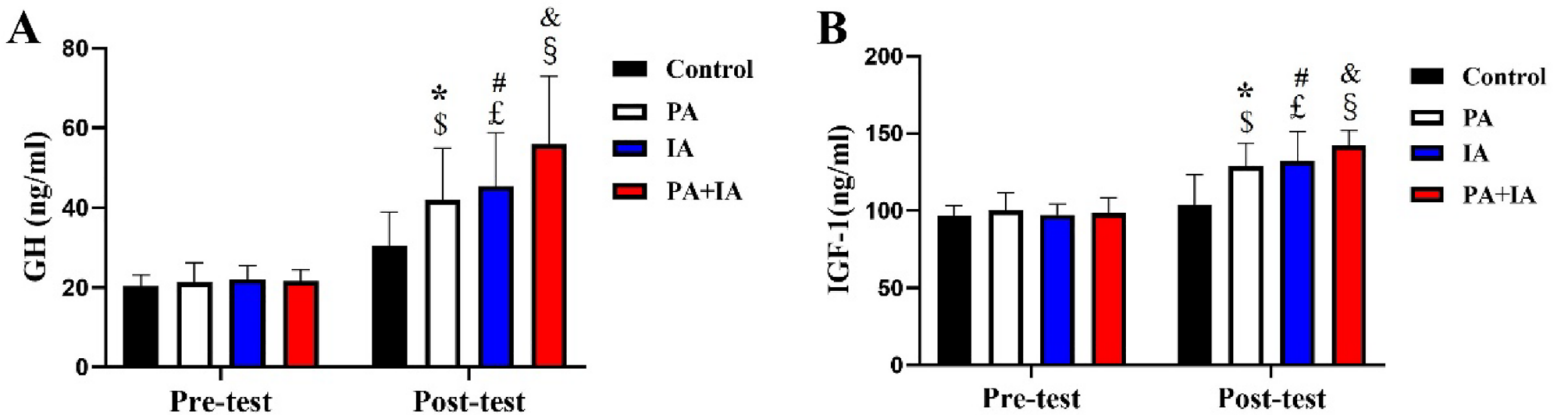
Effect of physical exercise program on GH-IGF-I axis in children. The results of GH (**A**) and IGF-I (**B**). *p < 0.05 represent Con vs. PA; ^#^p < 0.05 represent Con vs. IA; ^&^p < 0.05 represent PA + IA vs. Con. ^$^p < 0.05 represent Pre-test vs. Post-test in the PA group; ^£^p < 0.05 represent Pre-test vs. Post-test in the IA group ; ^§^p < 0.05 represent Pre-test vs. Post-test in the PA + IA group; N = 16–24/per group.

The variables of saliva GH and IGF-I concentrations in the pre-test and post-test stage can be found in the Fig. 5. Figure 5 demonstrated that saliva GH and IGF-I concentrations did not differ among four groups in the pre-test stage (*p* > 0.05). However, saliva GH and IGF-I concentrations significantly increased in the PA, IA, PA + IA groups than that in the Con group (p < 0.05), and the order is PA, IA, PA + IA.

We also learn by intra-group comparison that there was no significant difference of saliva GH and IGF-I concentrations between pre-test and post-test stage in the Con group (p > 0.05). Saliva GH and IGF-I concentrations were significantly increased of post-test stage in the PA group when compared with the pre-test stage (p < 0.05); this phenomenon also can be found in the IA, PA + IA groups (p < 0.05).

Base above, it showed that both physical and intellectual activity can effectively improve GH and IGF-I level in children, and the improvement effect of the two exercises together is more significant than that of the single exercise ways.

## Discussion

The purpose of the present study was to evaluate the effect of different aerobic exercise program on physical fitness and executive function in children. We hypothesized that both physical activity and intellectual activity can effectively improve physical fitness and executive function in children, and the improvement effect of the two exercises together is more significant than that of the single exercise ways. To test our hypothesis, we collected experiment information of children in each group at the pre-test and post-test stage, including body composition, physical fitness, executive function and GH-IGF-I concentration. Simultaneously, the following results will be discussed.

In this study, we first to explore the effect of exercise interventions on body composition in children. As we all know that early childhood is the most intensive period of body growth and development during the lifespan, their height and weight as well as other body shape index changes accordingly with the developing of skeletal systems^22,23^. A cross-sectional study with 423 children revealed that children who regularly participated physical exercise presented better body composition than inactive children, including higher lean body mass and height, lower body weight and fat mass index^24^. In contrast, Zhang M’s study directly revealed that there was no significant change observed of body shape index after 11 weeks physical exercise intervention in 6-year-old children^11^. Consist with this conclusion, our study also demonstrated that there were no significant differences of anthropometric measurements index before and after different exercise interventions between the two groups, such as height, weight, and BMI. We bold speculated that 16 weeks exercise interventions in our study is not sufficient to observe changes in body composition. Thus, we concluded that there was no significant effect of exercise interventions on body composition in children, but whether there are differences in other aspects of exercise still needs to be further explored.

Next, we also to explore the effect of different exercise interventions on physical fitness in children. The most widely used parameters of physical fitness in children at the primary level include cardiorespiratory fitness, muscular strength, motor fitness, global physical fitness^25,26^. Our results demonstrated that 16 weeks exercise interventions significantly improve physical fitness in children, including cardiopulmonary fitness, muscle strength, speed sensitivity, flexibility quality. Other’s study also confirmed by a large-scale study that the positive effect of exercise on physical fitness in children. The main findings of this article were that 8 to 10 years old children involved in sports clubs with regular exercise have higher physical fitness level than children not active in sports clubs^24^. Along with the global sports rapid development, the form and content of the exercise are also increasingly diverse. Exercise are no longer just about physical activities, but also include intellectual activities, such as painting^27^, international chess^28^, computer programming^29^, mindfulness training^30^. Our study was innovative to explore the variation of physical fitness when children received physical activity and intellectual activity at the same time. As result, we further demonstrated that all of the physical fitness parameters s were affected by physical activity or/and intellectual activity to some extent, as the effects of the activity intervention were the greatest when two exercise interventions were performed simultaneously.

Recent research suggests an interrelationship between physical fitness and executive function^31,32^. Physical fitness is closely related to brain health. Brain connectivity and executive function are directly and positively affected by physical fitness, eg. cardiopulmonary fitness, muscle strength, speed sensitivity, flexibility quality^33,34^. Therefore, we further to investigated the effect of different exercise interventions on executive function in children. Our study demonstrated that different exercise, including physical activity, intellectual activity, physical activity couple with intellectual activity, all can effectively improve the executive function in children. The result from a cross-sectional study generally highlighted a positive influence of physical activity on executive function in schoolchildren. This article also directly pointed out that executive function act as mediators of the association between physical activity and academic achievement^35^. Muntaner MA’s finding further provided the evidence that regular physical exercise leads to improvements in physical fitness and may support cognitive skills and academic performance in children aged between 9 and 13 years old^36^. And on this basis, we further demonstrated that the improvement effect of the two exercises together is more significant than that of the single exercise ways.

As is well-known that regular exercise improves physical fitness and executive function and is increasingly being undertaken on various forms of exercise^37,38^, but the neurobiological mechanisms underlying physical fitness and executive function have not been fully elucidated. As exercise impacts many of the endocrine system’s homeostatic functions, these interactions are especially important for the GH/IGF-I axis^39,40^. Exercise promotes anabolic components of the growth GH—IGF-axis in children^41^; on the contrary, the typical increase in circulating GH seen in response to exercise would be attenuated in children with disease, such as ADHD^42^, overweight, and obesity^43^. As expected, our results revealed that different types of exercise can effectively improve GH and IGF-I level in children, such as physical activity, intellectual activity, physical combine with intellectual activity. We found innovatively that the improvement effect of the two exercises together is more significant than that of the single exercise ways.

Our study was restricted by a small sample size and a relatively brief period of exercise intervention. As a result, a bigger sample size randomized controlled trial may provide more insight into the effect of different exercise on physical fitness and executive function. In addition, the neurophysiological evidences of different exercise interventions on physical fitness and executive function are further needed to be studied. Therefore, in the next step, ancillary neuroimaging or brain imaging studies of longitudinal exercise intervention may provide an opportunity to better understand the relation among physical fitness executive function and exercise.

## Data Availability

The datasets in this experiment are available from the first author (renyue 2021@cupes.edu.cn) on reasonable request.
